# Malaria and the Cuban Campaign

**Published:** 1898-07

**Authors:** Charles E. De M. Sajous


### Selections and Abstracts.

### MALARIA AND THE CUBAN CAMPAIGN.

In the spring of 1895 the writer witnessed the departure from Paris of the Two Hundredth Infantry for Madagascar. Beaming with anticipations of a glorious campaign, the young soldiers composing the regiment seemed capable of any physical effort; robust, agile, and merry, they appeared able not only to defy the efforts of any human foe, but also to remain invulnerable to the onslaught of a much more potent enemy, the noxious emanations of marsh lands and pools. The campaign was pushed with energy; but some months later every Parisian's heart was stirred to the utmost by the return of the same regiment, or, rather, what was left of it: a handful of gaunt, yellow "convalescents," several of whom were soon to follow the nine hundred and odd comrades who had preceded them in the other world. The human antagonist had been a myth; the subtile earth-and-water-born enemy had done it all.

The Spaniards have already said that their strongest allies would be the febrile diseases of Cuba: yellow fever and malaria. We have no reason to disagree with them, and, if the disastrous Madagascar campaign is taken as *theme*, it is because from the sanitary aspect there is a striking analogy between it and the Cuban campaign upon which we have just embarked. Geographically, Madagascar and Cuba may be called "sister isles"; the former is centrally traversed by the 22d degree of latitude south, the latter by the 22d degree of latitude north. Both lie to the southeast of the mainland; both are surrounded by warm ocean currents; both are paradises of fertility—studded with hot-beds of decaying vegetable matter, marshes, and swamps. When the resisting powers of the French and American forces are compared, they may be said to be very similar; the Foreign Legion of France is represented by our regulars; the French regiments of the line, made up of men between twenty-one and twenty-four years of age, about correspond to our volunteers, ranging in age from twenty-one to thirty-five, who compensate through their greater proximity to full development for the superior physical training of the French troops,—which, by the by, malaria in no way respects. As to the sanitary organization, the French medical corps was severely criticised by the lay press when the deplorable condition of the troops was made known; how far this was warranted it does not belong to us to state, but it can safely be asserted that a more loyal and better-versed corps of military surgeons can be found nowhere.

The fact remains, however, that if such results are possible with a medical corps organized on a permanent footing to satisfy the needs of a standing army of half a million men, to expect perfect service from a corps such as ours, elaborated for an army of twenty-three thousand men and suddenly increased to one of nearly two hundred thousand, is certainly paying Surgeon-General Sternberg a high tribute; one, indeed, which he truly merits.

The picture witnessed at Madagascar, however, can but suggest the possibility of similar experience in our case. A review of the main features of the French expedition may, therefore, prove instructive.

Among the multitude of statements published during the Madagascar campaign in connection with the high mortality among the troops, but three general factors seemed to be based on good ground: 1. A certain amount of friction between the army and navy, which indirectly caused vexatious delays and undue retention of troops in malarial districts. 2. Unwarranted subservience of all considerations bearing upon the health of the men to the needs of the expedition. 3. Inadequate application of prophylactic measures as a result of the first two factors.

We have no reason to believe that there exists any friction whatever between our army and navy. As to the absolute subservience of all hygienic considerations to the needs of the campaign, we surmise that no decisive step is taken by the Secretary of War when troops are to be transferred to malarious regions without due consultation with the Surgeon General, and that his advice is the primary ruling factor. As to the third, it is normally eliminated by the absence of the first two drawbacks. We can, therefore, feel that the conditions which have prevailed in Madagascar are not to be feared, *provided our means of prophylaxis are adequate.* But is this the case? Can we rely upon the preventive means dictated by prevailing views to efficaciously protect our troops? We must admit that our confidence becomes suddenly checked at this point. In the editorial offices of the *Annual* we peruse thousands of papers yearly, and we have learned to fear an exaggerated confidence in the teachings of experimental evidence. We have observed that, since bacteriology has invaded the very confines of pathology, experimental work has overshadowed clinical experience, and what the laboratory does not prove, what experiments do not show, is often refused in practice. If this tendency is not counteracted in the application of sanitary hygiene in the Cuban campaign, we might as well frankly admit that we firmly expect another Madagascar. Our purpose here is to urge that the distinguished members of our profession who are at present at the head of the army, navy, and marine-hospital bureaus be not unduly influenced by experimental evidence in the elaboration of the protective measures to be carried into effect.

Recently a prominent pathologist advanced the statement that at the present time there are only two theories as to the mode of transmission of malaria infection that are worthy of consideration, namely: that it occurs aerially, or by inoculation through the agency of suctorial insects. Such an assertion is quite acceptable when uttered as a theme for discussion before a medical society, but it is a very dangerous one to act upon when prophylactic measures are to be put into effect. True, the organism which is known to produce the fever called "malarial" in mankind has not been found outside of human blood; true, we have not been able so far to produce the disease experimentally by administering drinking-water from malarial places, but time has so frequently and so thoroughly contradicted positive assertions based on data of this kind, that negative experimental evidence thus obtained should count for naught when the least practical evidence to the contrary is obtainable. What we really know is that there are many factors, objective and subjective, which, acting, more or less conjointly, can give rise to malarial fever. Concerning two of these, aerial transmission and inoculation, we know something tangible; a third—the influence of drinking-water—does not occupy so advanced a position; a fourth—the predisposing influence of fatigue and heat—is still less understood; a fifth is hardly recognized: the effect of drenching either by wading across streams or by rain, or bathing in the waters of malarial districts, etc. That does not mean that we do not have evidence to sustain the belief that each one of these etiological elements may play an active part in the process; it simply indicates that the majority of them are not as yet understood. What do such negative experiments as those conducted by Celli and Zeri—the giving of marsh-water as beverage for a prolonged period to a large number of individuals—prove? Had their subjects been submitted to the exhausting effects of military life, long and perhaps forced marches; had they also inhaled "malaria" and used surface-waters from malarial pools as beverage, these investigators would probably have obtained different results. The coincidence of arduous field-service and fever has often been noticed; so great an authority on these matters as Laveran places transmission by drinking-water at the head of the list of pathogenic factors, and he has shown that malarious countries have been traversed with impunity by drinking only boiled water. Dr. Smart, of the United States Army, has witnessed the improved condition of troops suffering from malaria as soon as purer water-supplies had been afforded; our esteemed colleague, Dr. Shrady, editor of the *New York Medical Record*, has emphasized it.

While laying stress upon factors which may not have received the attention due them, our aim is not to undervalue those to which pathologists attribute the active role as intermediaries. Indeed, the Madagascar campaign affords a striking lesson as regards the importance of aerial transmission. To facilitate the transportation of baggage into the interior of the island, the French government had supplied its troops with metallic wagonettes, so constructed that they could be floated across streams. For one cause or another it was soon found that they could not be used, and the construction of a cross-country road was determined upon. The young soldiers were put to the task, and bravely and uncomplainingly they worked. Soon, however, the sick-list increased, and, by the time the road was finished, the mortality-list had become appalling. Why the military authorities permitted this when natives could be had to do the work it would be difficult to explain. To us, the lesson taught by this holocaust of victims is that under no circumstances should unacclimatized men be employed to work the soil. "Europeans must not attempt to work the ground in the intertropical regions," writes Medical Director Maurel of the French navy; "it is death to them, but it does not injure negroes or other natives, who should be secured for this purpose." The importance of aeriel transmission is further shown by Dr. Maurel's personal observations. In Africa he had spent nights tramping in the marshes without suffering the least inconvenience, while his assistants suffered from chills, merely because they did not seem to be able, literally speaking, to "keep their mouths shut." He absolutely refrained from talking so as to allow none of the miasmatic air to reach his lungs through the mouth, the nasal passages, as is well known, acting as a sterilizing apparatus through the destructive action of the nasal secretions upon an atmospheric organism.*

The bearing of atmospheric conduction upon the prophylaxis of malarial diseases shows itself in many other ways.

The accumulation of malaria near the surface accounts for the generally recognized fact that people who sleep on the ground-floor frequently suffer, while those who sleep in the upper stories remain unaffected. Hence the recommendation of hygienists to build houses on high places as far away as possible from gas-laden marshes and pools. The influence of accumulation on low levels was well shown recently by Mr. Stanley Kellett Smith, who related, in the *Lancet*, his observations during an exploration of the country west of Lake Nyassa. It was found that among the men on the march there was a close connection between fairly rapid alterations

---

*I would venture to suggest that we may have, in this prophylactic function of the nasal cavities, a clue to the manner in which the system becomes infected through the respiratory tract. As is well known, to work malaria soil before the sun is well up or after the sun sets is a source of great danger. The pathogenic element, whether it be organic or gaseous (*grundluft*), being widely dispersed through solar heat, the nasal mucus easily disposes of the comparatively few germs present—if such be the active factors. At night the miasmatic air lies low, the proportion of malarial agents is comparatively much greater, and enough pass the nasal cavities to infect the system. This would clear a potent impediment in the way of the air-conduction theory, namely: the fact that malaria is not transported by the winds. It seems plain that strong air-currents should also be able to cause a wide diffusion of malarial elements. This view also accounts for the fact that malaria does not behave after the manner of diseases due to the inhalation of dust. Malaria, on the contrary, is most active on sultry days, when there is no wind, and on days following rain, when the dust cannot rise.

of level and the occurrence of malarial manifestations. Whenever the route lay across level or gently sloping country fever was rare, but quick change from high veldt to low veldt and vice versa was always marked by a great increase in the number of attacks. Again, the inhabitants of the Pontine Marshes have learned by experience to sleep on platforms supported on stakes from twelve to fifteen feet high; a practice which is also resorted to in the malarious districts of Greece. These facts sufficiently sustain the advantage of camping troops upon the most elevated ground that can be reached, the tents being so placed as to have their rear face the dominant wind of the region. In bivouacking, hammocks—readily transportable by cavalry and artillery—hung as high as possible materially reduce the chances of malarial infection by holding the sleeper, if not entirely above, at least in a comparatively arrefied part of the malarial stratum.*

The evidence in favor of contamination by suctorial insects is no less strong, and the fact that the musquito can thus transmit the pathogenic element of malaria is gaining ground every day. Two years ago Manson produced, in the human subject, attacks of typical malaria by the imbibition of water inoculated from the bodies of musquitoes which had bitten persons suffering at the time from malarial fever. Draining off the soil causes mosquitoes to dissapear; they enter rooms and are most active at night, and they always fly near the ground, especially around pools. All these features thoroughly associate the mosquito with malarial agencies. According to Bignani, the insect deposits its eggs in water or in damp places; from the eggs are hatched larvæ, which, very voracious, devour everything they encounter, among other things the bodies of the dead mosquitoes and the envelopes from which they emerged. Then they pass into the state of nymphæ, from which emerge the young mosquitoes. During this long period of life in damp soil or in water, and especially in the state of larvæ, they impregnate themselves with malarial germs.

Emin Pasha, during his long sojourn in Africa, had, as his constant companion, his mosquito net. The overseers of estates and farms in the Roman Campagna, who less frequently suffer from the fever than their laborers, are especially careful to avoid the bites and stings of insects, particularly during sleep. In autumn the mosquitoes increase after the rains, and so does the fever; both diminish with distinct parallelism.

Practically, mosquitoes probably represent the most trying

---

*While traveling in tropical countries I found this method very satisfactory, although it was sometimes difficult to get in and out of the couches. When the trees are too far apart a strong rope can be used as support, each end of the rope being tied to one of the trees. Several hammocks can then be hung to the rope, end to end. To avoid the danger of falling, the edges of the hammock just above the center should also be attached to the rope above. Two large trees, twenty to twenty-five feet apart, and three pieces of strong Manila rope, placed side by side, can thus serve for ten men. A mosquito net passed over the rope and adjusted around the hammock affords adequate protection against mosquitoes, gnats, flies, etc.

foe one has to contend with in malarial districts, when rapid changes of camping-grounds are necessary; but fortunately, the other prophylatic measures required include the selection of high ground, located as far as possible from pools, etc., and, as musquitoes are absent, or, at least, not so numerous under such conditions, unless winds passing over swamps carry them, the men will frequently obtain moments of respite from their attacks. It is during marches which take in high and low ground, virgin forests, swamps, pools, and shallow streams that the little pests become intolerable, and any one who has tried under a tropical sun to cover head and hands with mosquito netting knows what Hades might be like; it is something like being stifled and parboiled at the same time. At night, however, netting can be advatageously employed in many ways, even when lying under tents, which seem in no way to prevent their entrance.*

As may be seen, there is solid ground for the belief that atmospheric conduction and inoculation by suctorial insects—the musquito being probably but one of the active parasites involved—are important—perhaps the most important—elements in this case; but what we wish to emphasize is that other factors, acting simultaneously with the main ones, may prove equally active; sufficiently so, indeed, to counteract prophylactic measures based upon a narrow view of the teachings of experimental work. Interesting in this connection is a report of one of our correspondents upon the methods employed by the Zulus to enable them to resist tropical diseases. Experience has taught them that in warm countries frequent ablutions, especially during active perspirations, debilitate the system, and that baths are frequently followed by malarial fever. Copious ablutions in their opinion "open the pores" and facilitate the ingress of the malarial poison; their aim, on the contrary, is to close these avenues for disease-germs, and to insure this they apply fat over the entire body. Our correspondent, Mr. Crooenberghs, during a long residence among the Zulus, determined to test the accuracy of these observations. The results he gives in the following words: "I had obtained this information from Antione d'Abbadie, of the Institute of France, and certainly found it applicable in South Africa. We both observed its teachings and lived. while all those among my companions who sought relief in the free use of water in Africa died. The three who had, like us, abstained from its use still live." The habit of anointing the body with fat as a prophylactic measure is not

*For campaigns in tropical climates, our tents appear to be very deficient. The ground over which they stand allows the "*grundluft*," or "ground-air," upon which German authors lay stress as a prominent etiological factor of malarial fevers, to freely contaminate the air; the interstices under the walls and flys permit the free ingress of reptiles and insects. These defects could be easily counteracted by adding to the tent a water-proof canvas base or "floor" sewn to the bottom of the perpendicular canvas ends and sides, and rising in front some inches above the ground under the flys. Such a tent could be closed in on all sides and would afford greatly increased protection against the most active disease-dealing agents nature supplies in the tropics, and enable the troops to profit, to a great degree, of the hours reserved for rest.

limited to the Zulus. A number of African tribes resort to it, and I am told that the blacks in some parts of Guiana and other portions of South America do likewise. Indirect sustaining evidence is also obtainable in the records of explorers.

Are we to infer that the malarial poison is actually absorbed by the skin, or should we attribute the symptoms to the contact during active diaphoresis with water of a lower temperature than that of the body, as is the case in any climate? This answer can only be given when we shall have ascertained the nature of the element through whose intermediary the system becomes infected. The malarial organisms have not been recognized outside the blood of human beings; we know nothing of the life-history of the supposed parasite acting as external factor; we do not even know what malaria-laden air is laden with. Is it a spore, a ferment, a toxin, a gas? We do know, however, that the skin does absorb medicaments and that by cataphoresis we can sometimes cause the taste of a drug applied externally to at once be perceived. Grouping several factors,—(1) the predilection of malarial miasm, whatever that may be, for water,—leading to the natural deduction that a pond, a rivulet passing through a malarial region, etc., is but a solution or a mixture of the infectious element or elements; (2) the reduction of resisting power—whatever that may be—induced in the individual exposed to fatigue; (3) the relaxation of the skin incident upon or following active perspiration,—we can but conclude that there is sufficient ground to warrant at least a strong suspicion that the Zulus are right, and that the precautions that experience has suggested to them should be followed, with due regard, of course, for habits of cleanliness which characterize Anglo-Saxon civilization. Let it be said, however, that we have in this very quality a source of increased danger. The first impulse of the men when nearing a lake, a pond, a river, after arduous marching under a broiling sun, will be to "plunge in" and by doing so, inhale, drink, absorb, and what not, the element of destruction. How can this be prevented? The answer is: strict discipline as regards the enforcement of sanitary measures emanating from the surgeon-general's office. Cuba is not malarious everywhere. The men will, as much as possible, be camped where malaria is positively known not to exist. Here, of course, the camp-routine may be followed. It is in regions known to be malarious or where the least doubt exists that steps should be taken to protect the men from the effects of their own recklessness. Here, even, there are opportunities which may be taken advantage of to satisfy their bathing proclivities. The Zulus avoid bathing except during heavy rains, but they take advantage of these whevever opportunity offers. After resting there is no reason why the men should not be allowed to do likewise. During dry weather, however, cleanliness should be obtained by other means. Whatever be the malaria-causing factor residing in water, utilization of all the means we know of would alone insure comparative safety. But the transportation of condensing apparatus for larger quantities of water would, of course, involve considerable difficulty. The next best procedure would be to boil the water; here, however, the chances that "ground air" would be eliminated would be slight. Accepting "ground air" as an active factor and not knowing its identity, chemical means can not come to our aid. We are reduced to the use of small quantities of water for cleansing purposes in miasmatic regions when rain-water is not procurable. Washing the body with a bowlful of the local water, provided carbolic-acid soap be used on the wash-cloth, will, as far as we know, destroy any organism that may be present, while the small quantity on the surface will contain so small a quantity of "ground air" as to eliminate any danger of contamination should this happen to be the noxious agent. The carbolic-acid soap will also tend to prevent the attacks of the smaller insects, such as fleas, lice, ticks, etc.

Drinking-water, as we have already seen, merits the greatest attention. In the British service this subject is considered as one of capital importance, but, unfortunately, the manner of satisfactorily supplying sufficient quantities of pure water is far from settled. Dr. R. H. Castellote, in the *Lancet* of February 12, 1898, published notes on the Niger-Soudan campaign of 1896, which not only emphasize this fact, but also give valuable details regarding the use of portable filters. Each man had been supplied. as had been the case in the Ashantee expedition, with a small pocket charcoal filter in a metal case. These were found handy, but they required very frequent cleaning and the "inspiratory power required to get a small result" caused a strong tendency among the men to discard their use. Pasteur-Chamberland filters also required too frequent cleansing and soon had to be abandoned owing to the destructive wear and tear of transportation. Altogether the question of portable filters in the tropics was found to be a difficult one. Dr. Castellote outlines the principal requirements that an expedition filter should present, and furnishes valuable hints based, of course, upon personal observation:

" A filter should be strong enough to stand the knocking about that things get from native servants and carriers; it should be capable of turning out a large quantity of water in a short time; should be easily taken to pieces for the component parts to be cleaned at frequent intervals (the amount of deposition on porcelain or charcoal is evidence of the quantity of suspended matter in the water at most places); to be useful at short halts on the march it should be easily set going and packed up again; and, lastly, it must be fairly portable.

"On the whole, probably one of the larger forms of Pasteur-Chamberland filters fitted with pump and with the body made of earthenware or galvanized iron, and securely packed in an outer jacket of wood or wicker-work would turn out enough water for twenty men in a short time and would answer most of the other requirements. The only point not satisfactory would be its portability. It would form a complete load for one carrier, and if of earthenware possibly would have to be carried between two.

"Whenever possible, undoubtedly the best and most certain way of purifying, or, rather, sterilizing, water is by boiling. This in itself is sufficient to make the water good and palatable for drinking purposes if it is naturally fairly clear; but if, as usually is the case, there is much suspended organic matter in it, this can be largely removed by straining through fine muslin or a clean pocket-handkerchief before boiling.

"Boiling was the process, on the failure of our filters, that we most generally relied on for getting a drink. Each white man carried a vulcanite water-bottle of about one and a half pints' capacity, covered with felt and fitted with padlock, which was filled up with boiled water, tea, etc., over night. But with thirsty men on a long day's march under a tropical sun it can readily be understood that one and a half pints of fluid did not go very far, and three or four hours generally saw most of the water-bottles empty. After that, one had either to exercise a great deal of self-control or risk drinking unprepared water whenever we came to it. It was not nice and it was not wholesome, but at all events it was wet, and I think the latter quality was the one which recommended itself to many. The vulcanite bottles require careful attention. In a short time, especially if used to carry tea, they acquire a musty, stale odor, which communicated a similar taste to the water. Hot water shaken up with a little clean sand soon disposes of this trouble. The felt coverings absorb a good deal of water, which, on exposure to the sun and wind by evaporation, renders the contents of the bottles very fairly cool. It was customary to dip them whenever we came to water." These are timely hints.

A few words concerning prophylactic measures addressed directly to the blood may not be out of place. That quinine merits, on the whole, the confidence of army surgeons as a potent prophylactic need not be emphasized; we positively know that the spores of the *plasmodivm malariæ* are extremely susceptible to its action, even when the proportion in the blood is minute. But we also know that it occasionally fails, *especially when general measures of prevention are not resorted to.* The conclusion imposed upon us, therefore, is that quinine should occupy an important position in the general prophylactic plan adopted, but that it should not alone be depended on; in other words, none of the other measures should be be neglected if its best effects are to be obtained.

What is the best salt to use, what is the quantity that should be imployed, and, lastly, what is the best time of the day to administer it to prevent untoward effects, gastric and general? Our late associate editor, Dr Dujardin-Beaumetz, having announced to the Societe de Therapeutique of Paris that the soldiers about to be sent to Madagascar were to carry with them, for prophylactic purposes, a considerable quanity of quinine, and that it would be desirable to know if one of the salts of that drug were preferable to another, and also which was the most suitable pharmaceutical form for administration, the society appointed a commission to investigate the subject. The results of their studies were embodied in the following conclusions; 1. The basic quinine hydrochlorate is to be preferred as a prophylactic measure, as being sufficiently soluble and containing the largest quantity of the alkaloid. It is also less irritating to the digestive passages than the sulphate. The hydrobromate should be employed in rebellious cases of fever in which the sulphate has failed. 2. The compressed preparations should be absolutely rejected; pills made with a soluble excipient or gelatin capsules seem to be the preparations of choice in preventive treatment. 3. Two pellets or gelatin capsules containing four grains of the hydrochlorate may be given daily; one in the morning and one in the evening. This quanity will be sufficient to maintain the organism continually under the influence of the quinine.

For the reasons already outlined, the prophylactic use of quinine was not systematically resorted to during the Madagascar expedition, but a review of the general literature on this subject has shown that the above conclusions coincide with the views of the majority of writers who speak from the standpoint of personal experience. Small doses are not reliable; large doses are apt to be hurtful. Four grains night and morning were found to do no harm, even if administered during long periods; provided the drug was given during meals. Administered in this manner, quinine will not even, according to Laveral, irritate the stomach. The author also recommends the hydrochlorate; but he states that it should not be taken with coffee, this beverage causing a portion of the quinine to be precipitated. Thin advises that it be given two days before the malarial district is reached.

In the preceding pages stress has been laid upon the fact that the experimentally demonstrated data at our disposal concerning the etiology of malarial fevers are insufficient to serve as reliable guides in the selection of the proper prophylactic measures to be used in a campaign so fraught with climatic dangers as that just begun; it has been suggested that, by using, in conjunction with these, the protective measures that explorers, expedition surgeons, residents of malarial districts, etc., have found effective,—although the *modus operandi* of these measures be still unexplained,—the efficiency of our life-saving efforts might be enhanced. Finally, it has been shown that a broad view of the question seemed to indicate that the fever-producing elements reached the system in various ways, and that it was only by counteracting simultaneously all the factors acting as intermediaries between cause and effect that we could hope for the best results.

Considered from this point of view, the following prophylactic measures, carried out simultaneously, become necessary in malarial districts to insure adequate protection:

1. To avoid contamination through the respired air and inoculation by insects:

Unacclimatized men, white or black, should not be employed for the digging of trenches, the erection of defences, or any other kind of work involving upturning of the soil. Natives should alone be utilized for this work.

High ground should be selected for camp-sites, windward, if possible, of any swamp, pool, stream, etc., that may be in the neighborhood.

The men should sleep as high above the ground as possible (not less than two feet and, if practicable, from twelve to fifteen feet) and be provided with mosquito netting.

While crossing the malaria-laden forests, glens, lowlands, swamps, etc., the men should be ordered to avoid talking.

2. To avoid contamination by water:

When water from malarial regions is alone available for drinking purposes, it should be filtered or, preferably, sterilized by boiling.

Bathing should not be permitted when water from a malarial region can alone be obtained, but washing of the body with such water is permissible, provided carbolic-acid soap be employed.

3. To prevent the development of malarial parasites in the blood:

Four grains of hydrochlorate of quinine should be administered morning and evening during meals as prophylactic, beginning two days before the malarious region is reached.

4. To conserve the general powers of resistance of the economy.

Regular and frequent periods of rest should intersperse long marches. Drenching and wading through streams should be avoided when possible. Varied and adequate food should be furnished.

The head should be so protected as to secure a mamimum amount of coolness under all degrees of temperature, a head-gear such as the solar tepe being furnished for this purpose.

It is obvious that the necessities of a military campaign greatly restrict possibilities, but the fact remains that the aim should be to do the best that can be done, all things considered. This will certainly be the case with such men as Drs. Sternberg, Van Reypen, and Wyman at the helm, and with the valued collaboration of Dr. Guiteras.

Speaking of malarial diseases, the *London Lancet* of March 12, 1898, very appropriately says: "We are impressed with the lamentable deficiency of exact knowledge which is still manifest notwithstanding all the industry of innumerable observers in the past fifty years." To recognize this is a great step forward; to recognize it in time before the lesson will have cost us a host of victims, as did to France during the Madagascar campaign, may, perhaps, do more for our cause than all the armies the country may bring to the front.—*Charles E. de M. Sajous, in Monthly Cyclopedia of Practical Medicine.*

